# Eukaryotic clamp loaders and unloaders in the maintenance of genome stability

**DOI:** 10.1038/s12276-020-00533-3

**Published:** 2020-12-18

**Authors:** Kyoo-young Lee, Su Hyung Park

**Affiliations:** Center for Genomic Integrity, Institute for Basic Science, Ulsan, Korea

**Keywords:** DNA synthesis, Checkpoints

## Abstract

Eukaryotic sliding clamp proliferating cell nuclear antigen (PCNA) plays a critical role as a processivity factor for DNA polymerases and as a binding and acting platform for many proteins. The ring-shaped PCNA homotrimer and the DNA damage checkpoint clamp 9-1-1 are loaded onto DNA by clamp loaders. PCNA can be loaded by the pentameric replication factor C (RFC) complex and the CTF18-RFC-like complex (RLC) in vitro. In cells, each complex loads PCNA for different purposes; RFC-loaded PCNA is essential for DNA replication, while CTF18-RLC-loaded PCNA participates in cohesion establishment and checkpoint activation. After completing its tasks, PCNA is unloaded by ATAD5 (Elg1 in yeast)-RLC. The 9-1-1 clamp is loaded at DNA damage sites by RAD17 (Rad24 in yeast)-RLC. All five RFC complex components, but none of the three large subunits of RLC, CTF18, ATAD5, or RAD17, are essential for cell survival; however, deficiency of the three RLC proteins leads to genomic instability. In this review, we describe recent findings that contribute to the understanding of the basic roles of the RFC complex and RLCs and how genomic instability due to deficiency of the three RLCs is linked to the molecular and cellular activity of RLC, particularly focusing on ATAD5 (Elg1).

## Genetic stability: DNA clamp proteins

The attachment and removal of clamp proteins that encircle DNA as it is copied and assist its replication and maintenance is mediated by DNA clamp loader and unloader proteins; defects in loading and unloading can increase the rate of damaging mutations. Kyoo-young Lee and Su Hyung Park at the Institute for Basic Science in Ulsan, South Korea, review current understanding of the activity of clamp loading and unloading proteins. They examine research on the proteins in eukaryotic cells, those containing a cell nucleus, making their discussion relevant to understanding the stability of the human genome. They focus particular attention on a protein called ATAD5, which is involved in unloading the clamp proteins. Deficiencies in ATAD5 function have been implicated in genetic instability that might lead to several different types of cancer.

## Introduction

Eukaryotic chromosomal DNA is duplicated by replicative DNA polymerases (Pols), Pol δ and Pol ε, which are tethered to a sliding clamp, proliferating cell nuclear antigen (PCNA). The DNA-encircling PCNA homotrimer increases the processivity of replicative DNA polymerases^1^. Repair DNA synthesis is the final step in excision repair and homologous recombination (HR) and is also carried out by DNA polymerases bound to PCNA. In addition to DNA polymerases, PCNA functions as a platform for recruiting many other proteins involved in different DNA transactions, such as lagging strand maturation, sister chromatid cohesion establishment, nucleosome reassembly, and DNA damage checkpoint activation. In addition, PCNA, when modified by ubiquitination and SUMOylation under specific cellular conditions, recruits proteins specialized in DNA damage tolerance pathways or anti-recombination activity^2^.

The closed ring-shaped PCNA homotrimer is abundant in the nucleus in its nucleoplasmic free form and DNA-encircling form; of these two forms, the latter participates in cellular activities regulating DNA metabolism. The two forms undergo transition through two processes: PCNA loading and unloading^3^. A closed PCNA homotrimer in the nucleoplasm is opened and loaded onto DNA by a clamp loader complex at a single-strand DNA/double-strand DNA junction (ssDNA/dsDNA) with a 3′-OH end. After PCNA completes this task, it is unloaded from the DNA by a clamp-unloading complex. To meet the high demand for PCNA during DNA replication, efficient cycling between nucleoplasmic PCNA and DNA-loaded PCNA through the loading/unloading process is critical.

In eukaryotes, another ring-shaped clamp, RAD9–RAD1–HUS1 (the 9-1-1 complex, Ddc1–Rad17–Mec3 in budding yeast), plays a role in ataxia telangiectasia-mutated (ATM)- and rad3-related (ATR)-mediated checkpoint activation after being loaded at damage sites^4,5^. Similar to the PCNA-loading process, the 9-1-1-loading process requires ring opening, followed by recruitment to DNA, which is performed by a specialized checkpoint clamp loader complex^6^.

PCNA is loaded onto DNA by the replication factor C (RFC) complex, which is a pentameric AAA^+^ ATPase complex composed of a large subunit, RFC1, and four small RFC proteins, RFC2, 3, 4, and 5^7–9^. In eukaryotes, three RFC-like complexes (RLCs) exist with overlapping, as well as distinct, cellular tasks (Table 1). Each RLC complex is composed of CTF18, ATAD5 (Elg1 in yeast), or RAD17 (Rad24 in budding yeast) as the large subunit and four small subunits, RFC2–5. CTF18 is stably associated with DCC1 and CTF8 and forms a heptameric complex with RFC2–5. The CTF18–DCC1–CTF8–RLC complex (hereafter referred to as CTF18-RLC) participates as a single entity in various DNA transactions, such as PCNA loading^10,11^, sister chromatid cohesion^12,13^, and DNA replication checkpoint activation^14,15^, although DCC1 and CTF8 are not essential for the PCNA loading/unloading activity of CTF18-RLC in vitro. ATAD5 (Elg1)-RLC is a primary PCNA unloader^10,16,17^. In contrast to the RFC complex and two RLCs, RAD17 (Rad24)-RLC functions as a 9-1-1 damage clamp loader^6^.

**Table 1:** A summary of the primary activities of the eukaryotic RFC complex and the three RLCs.

Biochemical and cellular activity	RFC		CTF18-RLC		RAD17 (Rad24)-RLC		ATAD5 (Elg1)-RLC	
	Human	Yeast^a^	Human	Yeast	Human	Yeast	Human	Yeast
PCNA loading	O	O	O	O	X	X	X	X
PCNA unloading	O (weak)^b^	O (weak)^b^	X	X^b^/O^c^	X	X	O	O
Cohesion establishment	Unknown	X	O	O	X	X	X	X
9-1-1 loading	X	X	X	X	O	O	X^b^	X^b^
Damage checkpoint	Unknown^d^	Unknown^d^	Unknown	X	O	O	X^e^	O^f^
Replication checkpoint	Unknown^d^	Unknown^d^	Unknown	O	O	X	Unknown	X^f^

^a^Yeast refers to budding yeast *Saccharomyces cerevisiae*.

^b^Based on in vitro data (ref. ^10,11^).

^c^Based on in vitro data (ref. ^11^).

^d^Since RFC1 is an essential protein.

^e^Ref. ^16^.

^f^Ref. ^18^.

Considering the multiple essential roles of PCNA in cellular processes, PCNA loading/unloading needs to be accurately regulated. The importance of PCNA loading/unloading regulation is strongly supported by the unviability of cells with deficient PCNA-loading processes and by the severe genomic instability in cells defective with PCNA unloading (in species from yeast to humans). In this review, we first describe the primary roles of eukaryotic clamp loaders/unloaders while focusing on recent findings that reinforce the original concept and help establish a composite understanding of the interrelationships between these basic roles. Next, building on recent findings, we outline the genomic instability observed in yeast and mammals with deficient clamp loaders/unloaders and describe in detail how genomic instability due to deficiency of the three RLCs is linked to their cellular activity, particularly focusing on ATAD5 (Elg1). We do not discuss PCNA modifications, such as SUMOylation and phosphorylation, or checkpoint regulation by Elg1-RLC in this review, since they are well described in other reviews, and their relevance to mammals is still being investigated^18,19^.

## Primary roles of the RFC complex and RLCs

### PCNA loading

The RFC complex is a primary PCNA loader. The ATP-bound RFC complex binds to and opens a PCNA homotrimer ring; the ring-opened RFC complex–PCNA intermediates bind to gapped or nicked DNA, and the binding is augmented by ATP binding. DNA binding of the intermediates leads to ATP hydrolysis, which triggers the release of PCNA from the RFC complex, followed by closure of the PCNA ring on the DNA. In vitro mutational studies have shown that the ordered hydrolysis of ATP in the RFC complex subunits is required for PCNA loading^7^.

PCNA is loaded primarily at ssDNA/dsDNA junctions with a 3′-OH end in different ways during DNA replication and repair. For lagging strand synthesis, the primer–template junction is repeatedly generated by DNA Pol α/primase. PCNA is loaded onto the primer–template junction, after which Pol δ synthesizes the discontinuous lagging strand. For leading strand synthesis, the first Okazaki fragment (passing over the origin of replication) is synthesized by Pol δ, followed by continuous leading strand synthesis performed by Pol ε^20,21^. In DNA repair pathways, the PCNA-loadable structures are provided by the 3′ ends in gaps formed during the incision/excision process or by the 3′ overhang that invades the sister chromatid during HR.

At the replication fork, it has been widely accepted that the leading and lagging strands are mainly replicated by Pol ε and Pol δ, respectively, based on strand-specific ribonucleotide incorporation mapping in yeast^22,23^. However, one report has suggested that Pol δ synthesizes both strands at the replication fork^24^.

Both yeast and human CTF18-RLC can load PCNA on gapped DNA with lower efficiency than the RFC complex but not on nicked DNA^10,11^. In the same in vitro system, RAD24-RLC and ATAD5-RLC cannot load PCNA. In both yeast and human cells, CTF18-RLC interacts with Pol ε via DCC1^25,26^. When complexed with nonsynthesizing Pol ε in this way, human CTF18–DCC1–CTF18 RLC can load PCNA more efficiently than pentameric CTF18-RLC^25,27^. A recent genome-wide analysis of PCNA occupation on the leading and lagging strands consistently showed that Ctf18-RLC preferentially loads PCNA on the leading strand, and that the RFC complex preferentially loads PCNA on the lagging strand^28^. This study, along with other studies, showed that interaction with Pol ε contributes to the recruitment of Ctf18-RLC to replication forks^28,29^. However, Ctf18 is not essential for bulk DNA replication and cannot substitute for Rfc1 deletion. Instead, PCNA loading by Ctf18 is known to be important for cohesion establishment and DNA replication checkpoint activation, which are discussed below.

### PCNA unloading

After completion of DNA synthesis during DNA replication and repair, PCNA is unloaded from DNA by the eukaryotic clamp unloader ATAD5-RLC (Elg1-RLC in yeast)^10,16,17,30^. The RFC complex can unload PCNA in vitro^9^, even though its activity is significantly lower than that of ATAD5-RLC^10^. It has been reported that yeast Ctf18-RLC can unload PCNA in vitro when the single-strand region of the DNA substrate is coated by heterotrimeric replication protein A (RPA)^11^. However, another recent study has shown that Ctf18-RLC catalyzes PCNA loading instead of PCNA unloading^10^. In addition, the physiological relevance of PCNA unloading by the RFC complex and CTF18-RLC has not yet been addressed. It has been shown that small-interfering RNA-mediated CTF18 depletion does not increase the amount of chromatin-bound PCNA in human cells^16^. The observed PCNA unloading by the RFC complex or Ctf18-RLC might represent the activity of subcomplexes of the small RFC subunits because PCNA unloading by RFC2, 3, 4, and 5 and RFC2, 5 subcomplexes has been reported in vitro^31^. However, ATAD5 (Elg1) is not an essential protein for cell viability^32,33^, and the amount of chromatin-bound PCNA is reduced after ATAD5-depleted cells enter the M phase^30^. Therefore, it is possible that the RFC complex or CTF18-RLC functions as a back-up PCNA unloader.

Similar to PCNA loading by the RFC complex, PCNA unloading by ATAD5-RLC requires a functional ATPase domain and interaction with small RFC subunits^10,16^. ATAD5 with mutations in the ATPase domain or RFC2–5-binding motif fails to reduce chromatin-bound PCNA accumulation in ATAD5-depleted cells. Single-molecule experiments have identified mechanical similarities and differences between PCNA loading and unloading processes^10^. In both processes, the binding of ATP to the RFC complex or ATAD5-RLC opens the PCNA ring. The RFC complex–PCNA intermediate binds to ssDNA/dsDNA junctions to initiate the PCNA loading process. However, ATAD5-RLC–PCNA intermediates are released from the DNA, and this step is not dependent on ATP hydrolysis. This suggests that ATP hydrolysis triggers the dissociation of PCNA from the bound RFC or ATAD5-RLC, followed by ring closure to complete the loading or unloading process, respectively. The following questions remain to be addressed in relation to the PCNA unloading process: (1) how and when do the ATAD5-RLC-PCNA intermediates dissociate from DNA and (2) how is the ATP hydrolysis of ATAD5-RLC triggered to separate PCNA from ATAD5-RLC.

During DNA replication, PCNA increases the processivity of tethered replicative DNA polymerases. In addition, PCNA recruits FEN1 and DNA ligase I for Okazaki fragment maturation and CAF-1 for nucleosome assembly^21,34,35^. Chromatin duplication is completed before PCNA unloading. In yeast, the depletion of the DNA ligase Cdc9 prevents the ligation of Okazaki fragments and results in PCNA accumulation on chromatin. The depletion of the histone chaperone CAF-1 or ASF1 in human cells delays PCNA unloading^17,36,37^. These results suggest that the PCNA-unloading process is tightly coordinated with DNA replication and nascent chromatin assembly. Pol δ inhibits RFC complex-mediated PCNA unloading in vitro^38^. FEN1, DNA ligase I, and Pol δ also inhibit ATAD5-RLC-mediated PCNA unloading to different degrees^10^. Therefore, it is possible that the PCNA-interacting replisome proteins compete with a PCNA unloader during DNA synthesis to prevent premature unloading of PCNA. Recently, another regulatory mechanism for PCNA unloading has been suggested. Bromodomain and extra-terminal domain (BET) family proteins are preferentially associated with chromatin enriched in histone H4 acetylated at lysine residues 5 and 12 and facilitate RNA Pol II-mediated transcription^39,40^. Kang et al. showed that after DNA replication, BET family proteins bind to nascent chromatin through interactions with newly synthesized histones that are acetylated at H4 lysine residues 5 and 12^41^. Nascent chromatin-bound BRD4 interacts with ATAD5 through its ET domain and inhibits the PCNA unloading activity of ATAD5-RLC to prevent premature PCNA unloading^41,42^.

### S-phase sister chromatid cohesion

In eukaryotes, replicated DNA is paired and held together upon synthesis until pair separation in mitosis. This sister chromatid cohesion is essential for the faithful transmission of replicated chromosomes during cell division^43,44^. Sister chromatid cohesion is achieved by the establishment of a large ring-shaped cohesin complex (which contains two coiled-coil subunits Smc1 and Smc3) concomitant with DNA replication. Cohesin loading onto chromosomes is assisted by the Scc2–Scc4 cohesin loader complex (NIPBL-MAU2 in humans) during the G1 phase. Cohesin release is facilitated by the Wapl-Pds5 dimer^45^. Cohesin is destabilized by Wapl and thus does not stay on DNA for a long time before chromosome duplication. During or after cohesin encircles the newly replicated sister chromatid, Smc3 acetylation by Eco1 acetyltransferase (ESCO1 and ESCO2 in humans) establishes stable cohesion by inhibiting Wapl-mediated cohesin destabilization^46–48^.

Several nonessential replisome proteins, such as Ctf4 and Chl1, contribute to cohesion establishment^43^. In addition, Ctf18-RLC is required for sister chromatid cohesion in yeast^12^. It has also been reported that human CTF18-RLC is important for sister chromatid cohesion and processive fork movement based on studies using *DCC1*-knockout retinal pigment epithelial cells, in which a marked reduction in CTF18 has also been reported^49^. In this report, SMC3 acetylation-mediated dissociation of WAPL-PDS5 was shown to be required for processive fork movement. The binding of EcoI acetyltransferase to PCNA is crucial for cohesion establishment during the S phase and for cell viability^50^. It has been suggested that during DNA replication, the PCNA mainly associated with Eco1 binding and subsequent Smc3 acetylation is loaded on the leading strand by Ctf18-RLC; the role of this PCNA is distinct from the primary role of the PCNA that is loaded by the RFC complex for DNA synthesis^28^.

The involvement of Elg1 in cohesion is not yet clearly defined. Smc3 acetylation is not reduced but actually slightly increased in *elg1∆* mutants^28^. This finding suggests that Elg1 does not have a role in cohesion establishment. In the same report, reduced Smc3 acetylation in *ctf18∆* mutants was rescued upon *elg1* loss. This finding suggests that the PCNA accumulated on the lagging strand upon *elg1* loss can support cohesion establishment in the absence of Ctf18-RLC-dependent PCNA loading on the leading strand. This result is consistent with the observation that *elg1* loss partially suppresses the cohesion defect and temperature sensitivity of *eco1-1* mutants^51^. *Elg1∆* mutants exhibit varying degrees of cohesion defect in several reports; however, in each report, the defect was milder than that observed in *ctf18∆* mutants^28,51,52^. It is likely that Elg1 has a role in cohesion establishment that is different from the well-established Smc3 acetylation-mediated mechanism.

During undisturbed DNA replication, PCNA is evenly distributed at both the leading and lagging strands^28^. Pol ε-mediated continuous leading strand synthesis is highly processive. Therefore, it remains to be elucidated how PCNA is frequently loaded onto leading strands. Because the ssDNA/dsDNA junction is required for PCNA loading by the RFC complex and CTF18-RLC, it is likely that at least the catalytic domain of Pol ε is temporally displaced from the 3′ end of the leading strand for CTF18-RLC-mediated PCNA loading. It has been proposed that Pol ε utilizes both the CDC45–MCM2–7-GINS (CMG) replicative helicase complex and PCNA as processivity factors to facilitate normal replication rates^21,53^. Tethering by the CMG complex might allow Pol ε to dissociate from the 3′ end of the leading strand but stays at the replication fork until leading strand synthesis restarts. The following remaining questions include: (i) if Pol ε is frequently halted, how is the new PCNA loaded after temporal Pol ε halting? (ii) How do extra PCNA molecules away from the replication fork function during cohesion establishment? (iii) How is PCNA unloading coordinated with cohesion establishment on leading strands?

### Checkpoint activation

When DNA damage sensors recognize damage, checkpoint pathways are activated to arrest the cell cycle and promote the DNA damage response and DNA repair. In human cells, ATM (Tel1 in yeast) and ATR (Mec1 in budding yeast and Rad3 in fission yeast) kinases are master regulators of major checkpoint pathways^5,54^. ATM is recruited to the broken DNA ends and then activated. ATR is activated when ssDNA levels increase due to various types of DNA damage and replication stress. ATR is recruited to RPA-coated ssDNA at the sites of DNA damage through its regulatory partner ATRIP (Ddc2 in budding yeast and Rad26 in fission yeast)^55,56^. Full activation of ATR activity at the damage sites requires TopBP1 (Dpb11 in budding yeast and Rad4 in fission yeast) and/or Ewing’s tumor-associated antigen 1 (ETAA1). TopBP1 is recruited to the damage site through its interaction with the 9-1-1 complex, and ATR is autophosphorylated^55,57^. RAD17-RLC (Rad24-RLC in yeast) loads the 9-1-1 complex at the dsDNA/ssDNA junctions of DNA damage sites in an RPA-dependent manner. The loaded 9-1-1 complex and RAD17-RLC bind with TopBP1, which is critical for TopBP1-mediated ATR activation. ETAA1 is recruited to RPA-coated ssDNA by directly binding to RPA and then activates ATR independently of TopBP1 but in parallel with TopBP1 recruitment^58–60^.

During DNA replication in budding yeast, stalled forks and DNA lesions, such as postreplication gaps and fork breaks, activate the Mec1–Rad53 (ATR-CHK2 in humans) checkpoint pathway but require different signal mediators, Mrc1 (CLASPIN in humans) and Rad9 (53BP1 in humans), respectively, for full Rad53 activation^61^. *Ctf18Δ* mutant cells are highly sensitive to replication stress induced by treatment with hydroxyurea, which depletes the nucleotide pool in cells^62^. Ctf18-RLC, but neither Rad24-RLC nor Elg1-RLC, is required for Mrc1-mediated DNA replication checkpoint activation upon hydroxyurea treatment^14,15^. Consistently, in *ctf18Δ* mutants, checkpoint activation in response to hydroxyurea-induced fork stalling is delayed and depends on the Rad9-mediated DNA damage checkpoint^14^.

Ctf18-RLC interacts with Pol ε via Dcc1^26^, and this interaction contributes to the recruitment of Ctf18-RLC to replication forks^28,29^. *Ctf18* mutants defective with Pol ε-binding were originally reported to be sensitive to hydroxyurea and defective in the replication checkpoint, as shown by reduced Rad53 phosphorylation^63^. On the other hand, two recent reports show that hydroxyurea sensitivity and reduced Rad53 phosphorylation are not observed in either Pol ε-binding-defective *dcc1* mutants or Dcc1-binding-defective *Pol ε* mutants^28,29^. However, the same paper showed that Rad52 foci, markers for recombination, and Psf1 phosphorylation, a marker for late origin firing, both of which are normally suppressed by checkpoint activation, are increased in Pol ε-binding-defective *ctf18* mutants treated with hydroxyurea. This suggests the importance of Ctf18-RFC recruitment to the leading strand for activation of the replication checkpoint^29^. Human CTF18-RLC interacts with Pol ε via DCC1^26^, but it is unknown whether this interaction is also important for checkpoint activation in human cells.

## The RFC complex and RLCs in cell viability and genomic stability

In eukaryotes, all five subunits of the RFC complex are essential for cell survival^33,64^. The other large subunits of RLCs, Rad24 (Rad17 in fission yeast and humans), Ctf18, and Elg1 (ATAD5 in humans), are not essential for cell viability in yeast^32,65^ or mammals^33^. Although these RLC genes are not essential for normal cell proliferation, a portion of human cancer cells depends on each gene for survival, according to genome-scale clustered–regularly interspaced–short-palindromic-repeat (CRISPR)–Cas9 screen data^33^.

*Rad24Δ* mutants display sensitivity to ultraviolet radiation and methyl methanesulfonate (MMS)^66^. *RAD17*-knockout HCT116 human colon cancer cells display defects in damage-induced CHK1 phosphorylation by ATR. Consequently, these cells are defective in mitotic and S-phase damage checkpoint functions, as seen in the case of yeast *rad24* deletion, and show chromosomal aberration and endoreduplication^67^. *Rad17*-knockout mice are embryonically lethal^68^. However, embryonic stem cells can be recovered from knockout mice; these knockout embryonic stem cells show DNA damage-dependent aberrant recombination and hypersensitivity to various DNA‐damaging agents.

Ctf18 was first identified as a suppressor of chromosome missegregation in budding yeast^9,19^. *Ctf18Δ* mutants display chromosome loss, elevated recombination frequency, sensitivity to MMS, and telomere mislocalization. Later, its roles in sister chromatid cohesion, checkpoint activation, and PCNA regulation were revealed in both yeast and mammals. *Chtf18* (mouse CTF18)-knockout mice are viable but display defects in meiotic recombination, leading to chromosome missegregation and ultimately an abnormal chromosome number^69^.

Elg1 was first identified as a suppressor of gross chromosomal rearrangements in budding yeast^19^. *Elg1Δ* mutants display gross chromosomal rearrangements, chromosome loss, an elevated recombination rate, telomere lengthening, a high mutation rate, and sensitivity to MMS^19^. In human ATAD5-depleted cells, there is an increase in spontaneous HR but a reduction in double-strand DNA break (DSB)-induced HR^70^. A recent paper showed that the centrosome is overduplicated upon ATAD5 depletion^71^. These findings suggest that ATAD5 (Elg1) is critical for maintaining genome stability. *Atad5* homozygous mutant mice are embryonically lethal^72^. *Atad5* heterozygote mutant mice exhibit genome instability and develop tumors^72^. Somatic ATAD5 mutations are found in human patients with endometrial cancer and intraocular melanoma^10,72^. In addition, a genome-wide analysis identified the *ATAD5* gene as a susceptibility locus for breast and ovarian cancers^73,74^. These observations suggest that ATAD5 functions as a tumor suppressor.

## Mechanisms by which ATAD5 (Elg1)-RLC maintains genomic stability

Most of the genomic instability observed in cells depleted of CTF18 or RAD17 (Rad24 in budding yeast) can be explained or inferred by their primary activity. In the case of ATAD5 (Elg1), considering that PCNA unloading is the final step after DNA synthesis and, in case of DNA replication, the final step after postreplication processes is completed, a defect in PCNA unloading cannot be easily linked to the severe genomic instability observed in ATAD5 (Elg1)-depleted cells. However, accumulating data suggest that accumulated PCNA or PCNA that remains on the DNA for a long time due to depletion of ATAD5 (Elg1)^16,17^ can be the primary cause of genome instability. Johnson et al. showed that alleviating PCNA accumulation by either disassembly-prone PCNA mutants or G2/M-specific Elg1 expression rescues genome instability, such as hyperrecombination, telomere lengthening, and MMS sensitivity, in *elg1∆* mutants^75^. Using site-specific mutations in Elg1 with different PCNA unloading activities, Shemesh et al. showed that DNA damage sensitivity and recombination rates correlate with the level of PCNA accumulation on DNA^76^. The effect of long-term PCNA residence on DNA on other processes is discussed below.

The C-terminal region of ATAD5, which contains the ATPase domain and the small RFC interaction domain, is sufficient for PCNA unloading in vitro and in cells^10^. The N-terminal region of ATAD5 interacts with many other proteins and these interactions are important for different activities regulating DNA metabolism. For example, ATAD5 interacts with the ubiquitin-specific protease 1 (USP1)-associated factor (UAF1) complex through the N-terminal domain and contributes to the PCNA deubiquitination process that terminates error-prone translesion synthesis (TLS)^77^. The biological importance of these protein–protein interactions is discussed below based on recent reports.

Taken together, timely PCNA unloading and proper protein binding or recruitment to replication forks by ATAD5-RLC contribute to many cellular activities regulating DNA metabolism, either alone or in concert, to maintain genomic stability.

### S-phase progression

S-phase progression is delayed in ATAD5 (Elg1)-depleted cells^16,78,79^. In human cells, it has been shown that this results from a slow DNA replication rate (measured by 5-ethynyl-2′-deoxyuridine incorporation into nascent DNA) but not checkpoint activation^16^. A ATAD5 mutant with defective PCNA unloading fails to restore the reduced DNA replication rate upon ATAD5 depletion^10^, which suggests that PCNA remaining on DNA might be the reason for the slow replication rate. However, DNA fiber experiments have revealed that there is no change in the replication fork speed or interorigin distance upon ATAD5 depletion^16^. A slow replication rate can result from defects in the formation of new replication factories upon capture of related proteins by accumulated PCNA due to the role of PCNA as an intrinsic scaffold. Many replication proteins are enriched on chromatin in ATAD5-depleted cells, and this accumulation is attenuated when PCNA is depleted^16^.

Two recent reports have suggested that PCNA retention on DNA can lead to defects in replication-coupled nucleosome assembly in *elg1∆* mutants. Gali et al. showed that postreplication nucleosome assembly, which is measured by the Okazaki fragment lengths and micrococcal nuclease sensitivity of newly replicated DNA, is defective in *elg1∆* mutants^80^. Janke et al. showed that transcriptional silencing at specific loci maintained by histone chaperones is defective in *elg1∆* mutants, and transcription is rescued upon the overexpression of the histone chaperone CAF-1^81^. In both reports, defects caused by *elg1* loss were not observed in the disassembly-prone PCNA background. This outcome suggests that the PCNA remaining on DNA physically inhibits nucleosome assembly or that histone chaperones required for nucleosome assembly are trapped by PCNA accumulated on DNA. Depletion of CAF-1 or ASF1 has been reported to reduce DNA replication rate^36^. Therefore, defects in postreplication nucleosome assembly can be another cause of slow S-phase progression in ATAD5 (Elg1)-depleted cells.

### Mismatch repair

The DNA mismatch repair (MMR) pathway recognizes and corrects base mispairs, insertions, and deletions generated during replication and that escape proofreading by replicative DNA polymerases^82,83^. PCNA participates in several steps during the DNA MMR process, including mismatch recognition by MSH2–MSH6 or MSH2–MSH3, strand discrimination, strand excision, and repair DNA synthesis. Other negative effects of PCNA accumulation on DNA metabolism have recently been reported in the MMR pathway^84^. The mutation rate is increased in *elg1∆* mutants, but this is not observed in a disassembly-prone PCNA or *msh2∆*/*msh6∆*-mutant background. In contrast, *pcna* mutants in which PCNA is overretained on DNA up to the G2/M phase display an increase in the mutation rate, regardless of Elg1 level. In addition, Msh6 accumulates on chromatin in a PCNA-interaction-dependent manner in *elg1∆* mutants. In human cells, chromatin-bound MSH2 levels are increased upon ATAD5 depletion and reduced upon simultaneous PCNA depletion^16^. This finding suggests that MMR defects also occur in ATAD5-depleted cells via a similar mechanism. Interestingly, the same report showed that the mutation rate also increases through MMR-independent but accumulated PCNA-dependent processes in *elg1∆* mutants^84^. Based on the results in human cells, one such possible candidate process has been identified as monoubiquitinated PCNA-directed error-prone translesion DNA synthesis^77^.

**Fig. 1:**
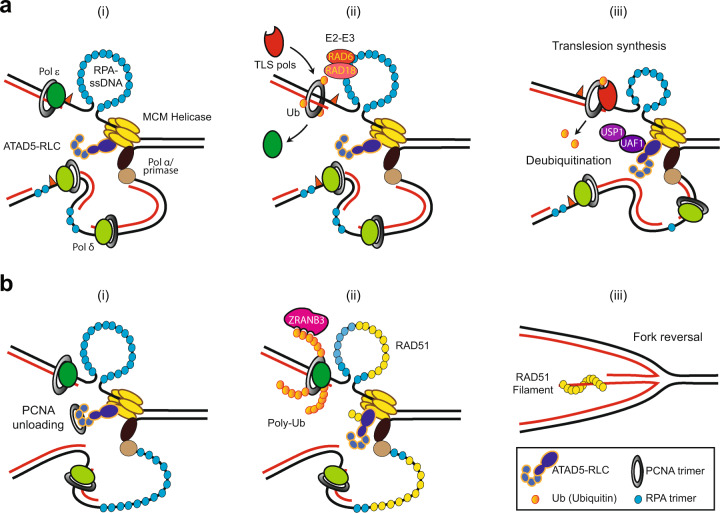
Graphical model of cellular mechanisms for preserving stalled fork integrity and the roles of ATAD5-RLC in this process. DNA lesions (orange triangle) and replication stress stall PCNA-tethered replicative polymerases physically (**a**, **i**) or chemically (e.g., nucleotide depletion) (**b**, **i**). This stalling causes the uncoupling of helicase and replicative polymerases, leading to the generation of a long single-strand DNA that is immediately coated by the trimeric RPA complex. **a**, **ii**, **iii** PCNA, monoubiquitinated by the E2–E3 RAD6–RAD18 complex, recruits translesion polymerases (TLS pols), and if the lesions are manageable by any of the TLS pols, the lesions are effectively bypassed. **a**, **iii** The USP1/UAF1 deubiquitinase complex removes ubiquitin from monoubiquitinated PCNA in an ATAD5 interaction-dependent manner, which reduces error-prone TLS-induced mutations. **b**, **ii**, **iii** Many DNA damage-inducing drugs lead to PCNA polyubiquitination by E2–E3 MMS2/UBC13–RAD5 protein. RAD51 recombinase and the translocase activity of HLTF and ZRANB3, which bind to the polyubiquitin chain of PCNA, cooperatively inducing fork reversal upon replication stress. ATAD5-RLC unloads PCNA in a timely manner and subsequently facilitates the recruitment of RAD51 to stalled forks, a process mediated by a replication stress-enhanced interaction between the two proteins. The RAD51 filament protects the reversed fork from nucleolytic attacks (**iii**).

PCNA is monoubiquitinated by the E2–E3 RAD6–RAD18 complex or by the CRL4^Cdt2^ ubiquitin ligase complex when a replication fork is stalled by DNA lesions or intrinsic replication blocks^85,86^. The resulting monoubiquitinated PCNA recruits error-prone TLS polymerases to bypass the DNA lesion (Fig. 1a). This potentially mutagenic TLS activity needs to be minimized in unperturbed cells and terminated immediately after lesion bypass by TLS polymerases. TLS termination is carried out by ubiquitin-specific proteases USP1 and/or USP10^87,88^. UAF1 is required for the optimal activity and protein stability of USP1^89^. ATAD5 interacts with UAF1, and this interaction is important for USP1-mediated PCNA deubiquitination (Fig. 1a)^77^. Consequently, in ATAD5-depleted human cells, the level of monoubiquitinated PCNA increases without exogenous DNA damage, and the mutation frequency is increased, as indicated by SupF assay^77^. Collectively, dysregulated MMR and translesion DNA synthesis can contribute to increased mutations in *elg1∆* mutants and ATAD5-depleted cells.

### Fork reversal

When DNA damage slows or stalls the progression of the replication fork, template DNA and nascent DNA reannealing can generate a four-way junction structure in a process referred to as fork reversal (Fig. 1b, iii), which can stabilize the replication fork^90^. RAD51 recombinase is important for both fork reversal and the stability of reversed forks^91–93^. In addition, three translocases, ZRANB3, SMARCAL1, and HLTF, have been shown to have fork reversal activity in vitro; of these proteins, ZRANB3 and HLTF have been shown to display this activity in cells^94–96^.

Damage-induced lysine 63 (K63)-linked PCNA polyubiquitination mediates the error-free DNA damage tolerance pathway through template switching. Production of a polyubiquitin chain for en bloc chain transfer to PCNA at K164 or sequential addition of ubiquitin to monoubiquitinated PCNA at K164 is mediated by the cooperative activity of the MMS2/UBC13 ubiquitin-conjugating dimer and either of the two yeast Rad5 ubiquitin ligase homologs, SHPRH and HLTF, or a currently unidentified E3 ligase^85,97–100^. The requirement of K63-linked PCNA polyubiquitination and polyubiquitinated PCNA interaction with ZRANB3 for damage-induced fork reversal suggests that fork reversal is closely linked to template switching^95,101,102^.

ATAD5 directly interacts with RAD51 through the N-terminal domain of ATAD5^103^. ATAD5 facilitates RAD51 recruitment to stalled forks by replication stress-enhanced protein–protein interactions (Fig. 1b)^103^. ATAD5 also removes PCNA from stalled forks in a timely manner for RAD51 recruitment. Consistently, ATAD5 depletion inhibits the deceleration of fork progression and reduces the native 5-bromo-2ʹ-deoxyuridine signal upon replication stress, which suggests inhibited fork reversal. This effect eventually leads to increased genomic instability, both in cells and mice undergoing replication stress. Since ATAD5 depletion increases the abundance of monoubiquitinated PCNA on DNA^77^, polyubiquitinated PCNA can also be increased in ATAD5-depleted cells. Therefore, conceptually, facilitated fork reversal by increased PCNA polyubiquitination through ZRANB3 recruitment might be expected; however, the actual observations are different in ATAD5-depleted cells^103^. This finding suggests that the RAD51 recombinase and the ZRANB3 translocase can cooperate to drive fork reversal, but depending on the type of genotoxicity, one of these proteins may be dominant. ZRANB3-depleted cells display higher sensitivity to camptothecin compared to other drugs, which suggests a high demand for ZRANB3 for processing camptothecin-induced DNA damage^102^.

### R-loop regulation

R-loops are reversible nucleic acid structures that feature a DNA/RNA hybrid and the resulting nonhybridized ssDNA^104,105^. R-loops are formed temporarily to regulate many cellular processes. However, persistent R-loops make the genome vulnerable to DNA damage due to exposure of ssDNA regions. R-loops can be formed during DNA replication when replicative forks collide with transcriptional machinery. Consistently, replication stress due to nucleotide depletion or DNA polymerase inhibition increases R-loop formation^106^. Transcription–replication conflicts interfere with replication fork progression, resulting in potential threats to genome stability^104^.

**Fig. 2:**
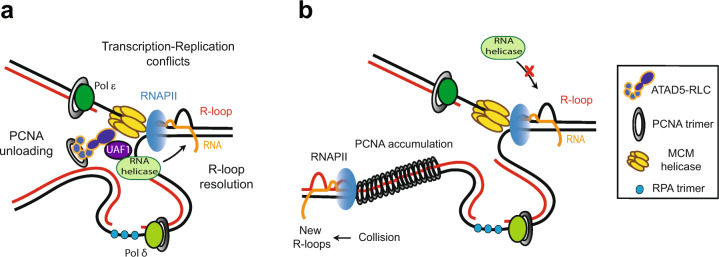
Graphical model for R-loop regulation by ATAD5-RLC and ATAD5/UAF1-interacting DNA/RNA helicases. **a** During normal replication, ATAD5-RLC and ATAD5/UAF1-interacting DNA/RNA helicases migrate with a replication fork. ATAD5/UAF1-interacting DNA/RNA helicases resolve R-loops ahead of the replication fork and facilitate replication fork progression. Upon replication stress, which increases transcription–replication conflicts and unscheduled R-loop formation, additional DNA/RNA helicases are recruited to the replication fork, which resolves R-loops to ensure faithful replication fork progression. Under both normal and replication stress conditions, the recruitment of helicases at the replication fork is dependent on the ATAD5/UAF1 interaction. **b** In ATAD5-depleted cells, reduced R-loop resolution by ATAD5/UAF1-interacting DNA/RNA helicases leads to defects in replication fork progression. In addition, PCNA and its interacting proteins accumulated on lagging strand DNA behind the forks collide with transcription machinery, which consequently increases R-loop formation at the collision site.

Recently, dual roles have been suggested for ATAD5-RLC in R-loop regulation during DNA replication (Fig. 2)^107^. ATAD5-RLC prevents the generation of new R-loops behind replication forks by unloading PCNA. Without ATAD5-RLC, PCNA accumulates and persists on the lagging strand DNA for a long time, causing a collision with transcription machinery, which can lead to R-loop formation. In addition, ATAD5 recruits DEAD/DExH-box DNA/RNA helicases at the replication forks through UAF1-mediated protein–protein interactions. Under replication stress, the same DNA/RNA helicases are additionally recruited to the replication forks in a process that is also dependent on helicase interaction with ATAD5/UAF1. These recruited DNA/RNA helicases then resolve R-loops under normal and replication stress conditions and facilitate replication fork progression.

### DNA double-strand break repair

DSB is one of the most dangerous types of DNA lesions. Nonhomologous end joining (NHEJ) and HR are the two primary double-strand DNA break repair (DSBR) pathways, while microhomology-mediated end joining and single-strand annealing are back-up mechanisms. A primary determinant for the pathway choice between NHEJ and HR is end resection, which predominantly occurs during the S/G2 phase to generate a long tract of 3′-OH ssDNA. End resection is required for forming the ssDNA-coated RAD51 filament that invades the homologous sister chromatid during HR.

The first evidence for the role of Elg1 (ATAD5) in DSBR was found because of the sensitivity of *elg1∆* mutants to phleomycin, a compound that generates DSBs, and reduced damage-induced recombination repair in *elg1∆* mutants^108^. The same report also showed that Elg1 is recruited to DSB sites independent of Rad52 (a key factor for HR in yeast) and that repair DNA synthesis but not PCNA recruitment is slightly defective upon *elg1* loss. In human cells, DSB-induced HR frequency is also reduced upon ATAD5 depletion^70^. A recent report showed that ATAD5-depleted cells are sensitive to MMS, bleomycin, and camptothecin, all of which can generate DSBs^109^. Consistent with the requirement of poly(ADP-ribose) polymerase (PARP)-mediated repair for the survival of HR-deficient cells^110,111^, ATAD5-depleted cells are highly sensitive to PARP inhibitors. Collectively, the positive role of ATAD5 (Elg1) in HR is evident, but the molecular mechanism remains to be studied. The Rad52-independent recruitment of Elg1 to DSB sites in yeast and the rapid localization of RFC complex proteins and PCNA to DSB sites in human cells suggest the involvement of ATAD5 (Elg1) in the early steps of HR^108,112^.

In addition to S-phase sister chromatid cohesion formed during DNA replication, genome-wide cohesion is generated by DSBs in both yeast and human cells^113,114^. In both human and yeast cell lines, damage checkpoint kinase-mediated phosphorylation and subsequent Eco1-mediated acetylation are required; the targets for both these protein modifications are Scc1 in yeast and SMC3 in human cells^115^. It is not clear whether the CTF18-RLC and PCNA are also involved in DSB-induced cohesion as they are in S-phase sister chromatid cohesion. However, a few reports support this possibility. Ogiwara et al. showed that Ctf18 is recruited to DSB sites; *ctf18∆* mutants are sensitive to DSB-inducing drugs, and damage-induced recombination between sister chromatids and between homologous chromosomes is defective in the same mutants^116^. In addition, it has been reported that PCNA moves rapidly to DSB sites even when RFC1 is depleted in human cells^112^, which also suggests that Ctf18-RLC serves as a PCNA loader at DSB sites.

## Concluding remarks

To date, elaborate efforts have elucidated many aspects of eukaryotic clamp loaders and unloaders, such as their basic biochemical and cellular activities and the molecular mechanism of genomic instability when their activities are diminished. As described in this review, recent findings have increased our composite understanding of how RFC/RLCs cooperatively ensure genomic integrity by spatiotemporally regulating PCNA, in addition to properly recruiting the necessary factors. However, it is still unclear how the knockout phenotypes in mice, especially embryonic lethality and tumor incidence in *ATAD5*-knockout mice, are related to the molecular and cellular activities of these proteins. In addition, several phenotypes, such as lengthened telomeres, a high recombination rate, and sister chromatid exchange in ATAD5 (Elg1)-depleted cells, require mechanistic explanations to understand how PCNA accumulation on DNA leads to these defects.
